# Glaucomatous visual fields and neurocognitive function are independently associated with poor lane maintenance during driving simulation

**DOI:** 10.1186/s12886-020-01682-9

**Published:** 2020-10-20

**Authors:** David E. Anderson, John P. Bader, Emily A. Boes, Meghal Gagrani, Lynette M. Smith, Jideofor K. Ndulue, Sachin Kedar, Vikas Gulati, Deepta A. Ghate, Matthew Rizzo

**Affiliations:** 1 Department of Ophthalmology & Visual Science, University of Nebraska Medical Center, 985540 Nebraska Medical Center, Omaha, NE 68198-5540 USA; 2 Department of Neurological Sciences, University of Nebraska Medical Center, Omaha, USA; 3 Department of Ophthalmology, University of Kentucky College of Medicine, Lexington, USA; 4 College of Medicine, University of Nebraska Medical Center, Omaha, USA; 5 Department of Biostatistics, University of Nebraska Medical Center, Omaha, USA

**Keywords:** Glaucoma, Visual field, Cognition, Executive function, Driving, Simulation, Vehicle control, Quality of life

## Abstract

**Background:**

Driving simulators are a safe alternative to on-road vehicles for studying driving behavior in glaucoma drivers. Visual field (VF) loss severity is associated with higher driving simulator crash risk, though mechanisms explaining this relationship remain unknown. Furthermore, associations between driving behavior and neurocognitive performance in glaucoma are unexplored. Here, we evaluated the hypothesis that VF loss severity and neurocognitive performance interact to influence simulated vehicle control in glaucoma drivers.

**Methods:**

Glaucoma patients (*n* = 25) and suspects (*n* = 18) were recruited into the study. All had > 20/40 corrected visual acuity in each eye and were experienced field takers with at least three stable (reliability > 20%) fields over the last 2 years. Diagnosis of neurological disorder or cognitive impairment were exclusion criteria. Binocular VFs were derived from monocular Humphrey VFs to estimate a binocular VF index (OU-VFI). Montreal Cognitive Assessment (MoCA) was administered to assess global and sub-domain neurocognitive performance. National Eye Institute Visual Function Questionnaire (NEI-VFQ) was administered to assess peripheral vision and driving difficulties sub-scores. Driving performance was evaluated using a driving simulator with a 290° panoramic field of view constructed around a full-sized automotive cab. Vehicle control metrics, such as lateral acceleration variability and steering wheel variability, were calculated from vehicle sensor data while patients drove on a straight two-lane rural road. Linear mixed models were constructed to evaluate associations between driving performance and clinical characteristics.

**Results:**

Patients were 9.5 years older than suspects (*p* = 0.015). OU-VFI in the glaucoma group ranged from 24 to 98% (85.6 ± 18.3; M ± SD). OU-VFI (*p* = .0066) was associated with MoCA total (*p* = .0066) and visuo-spatial and executive function sub-domain scores (*p* = .012). During driving simulation, patients showed greater steering wheel variability (*p* = 0.0001) and lateral acceleration variability (*p* < .0001) relative to suspects. Greater steering wheel variability was independently associated with OU-VFI (*p* = .0069), MoCA total scores (*p* = 0.028), and VFQ driving sub-scores (*p* = 0.0087), but not age (*p* = 0.61).

**Conclusions:**

Poor vehicle control was independently associated with greater VF loss and worse neurocognitive performance, suggesting both factors contribute to information processing models of driving performance in glaucoma. Future research must demonstrate the external validity of current findings to on-road performance in glaucoma.

## Background

Glaucoma, the second leading cause of blindness in the world [1], is an optic neuropathy characterized by optic disc cupping, retinal nerve fiber layer (RNFL) thinning, and progressive vision loss that starts in peripheral visual fields. Glaucoma patients are often unaware that their visual field loss restricts peripheral object detection [2–4] and real-world scene perception [5]. Visual field loss severity is associated with higher motor vehicle crash rates [6, 7], which are at least 4.2 times higher in patients diagnosed with moderate-to-severe glaucoma relative to healthy comparisons [8]. Neurocognitive performance, by contrast, is a strong predictor of driving safety [9, 10] in healthy drivers that remains understudied in glaucoma drivers [8, 11]. Efforts to mitigate increased motor vehicle crash rates in glaucoma drivers will require understanding the interaction between visual field loss and neurocognitive performance in this at-risk population.

Driving simulators are a safe and externally valid alternative to on-road vehicles for studying driving behavior in at-risk populations [12]. Although there are limitations to comparing driving performance across simulated and real-world environments, due in part to computational constraints on replicating the complexity of reality and the extent to which drivers suspend disbelief in a simulator, driving performance is comparable across platforms. For example, previous driving simulation studies show higher crash frequency in glaucoma drivers relative to healthy drivers [13, 14] that is predicted by degree of visual field loss [13], which is in accordance with previous on-road driving studies [6–8, 15]. In a population-based study of state motor vehicle crash records [6], Kwon and colleagues showed that binocular visual field loss in upper, lower, and left visual fields – but not right visual fields – were associated with higher crash rates. During a computer-based hazard perception task that induced simulated gaze-contingent visual field loss in healthy volunteers [16], Glen and colleagues showed that hazard detection speed was slower during conditions with, relative to without, visual field loss, where significantly slower speeds were observed in superior, relative to inferior, visual field loss conditions. Together, these studies provide converging evidence across simulated and real-world platforms to suggest that patterns of visual field loss are associated with increased crash risk, which may be explained by an inability to rapidly detect upcoming roadway hazards.

Mechanisms of how glaucomatous visual field loss contributes to predictors of increased crash risk, such as poor vehicle control [17], remain unclear. One possibility is that peripheral visual field loss causes poor lane boundary tracking and, by consequence, poor lane maintenance and vehicle control. In an on-road driving study, Bowers and colleagues found that subjective qualitative ratings of lane maintenance errors by a driving instructor were higher in glaucoma drivers with more restricted binocular visual fields [11]. Useful field of view (UFOV) divided attention performance also predicted lane maintenance errors under concurrent cognitive loads (i.e. merging, curve taking). Critically, however, interactions between binocular visual field loss and divided attention on lane maintenance errors, as well as their association with global neurocognitive performance, were unexplored. To our knowledge, current studies have yet to evaluate how binocular visual field loss and global neurocognitive performance interact to predict quantitative measures of lane maintenance in the driving simulator environment. Prior studies that have included neurocognitive measures in their design relied on dementia screening tools to adjust for mental status in their models without describing statistical contributions of mental status to their reported results [6, 8]. Thus, parsing out independent contributions of binocular visual field loss and pre-clinical variability in neurocognitive performance to predictors of increased crash risk, such as poor vehicle control, remain unexplored.

In the current pilot study, we sought to understand the relationship between binocular visual field loss, global neurocognitive performance, and quantitative measures of motor vehicle control (e.g. lane maintenance) in a driving simulator study of legally-licensed and actively driving glaucoma patients. To this end, we calculated binocular visual field parameters by integrating monocular Humphrey visual fields, assessed performance on the Montreal Cognitive Assessment (MoCA), and quantified both lateral and longitudinal vehicle control measures in glaucoma patients and suspects completing simulated driving scenarios. Given that vision precedes neurocognitive functions in models of the information processing pathway [18], study results will be useful for determining whether binocular visual field loss explains independent or overlapping sources of variability in driving performance in glaucoma drivers.

## Methods

### Patients

Subjects with glaucoma and glaucoma suspects were recruited from the Truhlsen Eye Institute at the University of Nebraska Medical Center (UNMC) in Omaha, NE. Glaucoma suspects [19] had normal visual fields; glaucoma patients had a range of visual field defects. Our design included glaucoma suspects as a control group for glaucoma patients because they present with the same pathophysiologic risk factors for glaucoma without visual field loss, allowing study results to be attributable specifically to visual field loss per se. All had corrected visual acuity of at least 20/40 in each eye and were experienced visual field takers with at least three stable (reliability indices better than 20%) visual fields over the last 2 years. Subjects were excluded if they had a diagnosis of neurological disorder or neurocognitive impairment so that we could focus on normal variability in neurocognitive performance. This study was approved by the UNMC institutional review board.

### Clinical characteristics

#### Visual fields

Visual field testing was performed in the ophthalmology clinic using the 30–2 or the 24–2 SITA standard strategy in automated perimetry (Humphrey Field Analyzer II-I (HVF), Carl Zeiss Meditec Inc) as part of routine clinical care prior to recruitment for the study. HVF assessed the central 48 (30 degrees nasally) and 60 degrees visual field of view in the 24–2 and 30–2 strategies, respectively. Mean deviation (MD) and visual field index (VFI) estimates the unimpaired proportion of visual field, ranging between 0% (fully impaired) to 100% (fully intact). HVF-VFI estimates were acquired from Humphrey Visual Field Analyzer.

Binocular VFI values were estimated using procedures that expand upon previous derivations of integrated visual fields [20] by: (1) deriving binocular threshold value estimates from monocular threshold values to more closely map VFI calculations; and (2) taking into account asymmetries in the distribution of neuronal resources in visual cortex across the visual field. First, we applied the Best Location Algorithm [21] to derive total deviation (TD) estimates for a theoretical binocular visual field (*bTD*_*i*, *j*_) estimate for each tested horizontal (*i*) and vertical (*j*) position within the HVF as:

$$ {bTD}_{i,j}={bTV}_{i,j}-\overline{bTV_{i,j}}, $$

where *bTV*_*i*, *j*_ is observed binocular threshold value (TV) and $\overline{bTV_{i,j}}$ is expected binocular TV. *bTV*_*i*, *j*_ and $\overline{bTV_{i,j}}$ were calculated as:

$$ {bTV}_{i,j}=10\ {\log}_{10}\sqrt{{\left({10}^{\frac{lTV_{i,j}}{10}}\right)}^2+{\left({10}^{\frac{rTV_{i,j}}{10}}\right)}^2}, $$

$$ \overline{bTV_{i,j}}=10\ {\log}_{10}\sqrt{{\left({10}^{\frac{lTV_{i,j}-{lTD}_{i,j}}{10}}\right)}^2+{\left({10}^{\frac{rTV_{i,j}-{rTD}_{i,j}}{10}}\right)}^2}, $$

where *lTV*_*i*, *j*_ and *rTV*_*i*, *j*_ are left and right HVF TV estimates, respectively, and *lTD*_*i*, *j*_ and *rTD*_*i*, *j*_ are left and right HVF TD estimates, respectively, for each location *i* and *j*. Note that these calculations transform TV estimates from logarithmic to non-logarithmic values before applying the quadratic summation equation, and subsequently transforms back into the native logarithmic space.

Next, *bTD*_*i*, *j*_ estimate were transformed into sensitivity estimates (*S*_*i*, *j*_) that ranged from 0 to 100, as:

$$ {S}_{i,j}=\left\{\begin{array}{c}100-\left(100\frac{\left|{bTD}_{i,j}\right|}{\overline{bTV_{i,j}}}\right),\kern0.5em {bTD}_{i,j}<0\\ {}100,\kern0.5em {bTD}_{i,j}\ge 0\end{array}\right. $$

Finally, binocular VFI estimates were derived by calculating the linear sum of *S*_*i*, *j*_ and a physiologically accurate weighting matrix (*W*):

$$ VFI=\frac{\sum {S}_{i,j}\ast {W}_{i,j}}{\sum 100\ast {W}_{i,j}} $$

Critically, matrix *W* gives higher importance to central and paracentral points as compared to the peripheral points divided into five concentric rings of increasing eccentricity and decreasing weight magnitude [22]. Specifically, the central four points were allotted a weight of 3.29, and with increasing eccentricity the weights decreased from 1.28, 0.79, 0.57 to 0.45.

#### Retinal imaging

Cirrus HD-OCT (Carl Zeiss Meditec Inc.) acquired scans of the optic nerve using the Optic Disc Cube protocol. Good quality scans with appropriate centering, clear images, and signal strength > 6 were included in analyses. We recorded RNFL global thickness measurements.

#### Neurocognitive assessment

Due to the exploratory nature of this study, we sought to evaluate global neurocognitive performance, rather than a comprehensive battery of neurocognitive assessments, to identify preliminary interactions between binocular visual fields and neurocognitive performance on simulated driving performance. We assessed global neurocognitive performance data using the MoCA, a brief screening tool that assesses 7 neurocognitive domains: visuospatial and executive functioning, object recognition, verbal memory, attention, verbal fluency, abstract reasoning, and orienting. MoCA is reliable and valid tool that is widely used for screening normal neurocognitive function [23–25].

#### Visual quality of life

Visual quality of life was assessed using the National Eye Institute (NEI) Visual Function Questionnaire (VFQ). Patients self-administered the questionnaire and asked the experimenter questions as needed. The NEI-VFQ has good reliability and validity for assessing the influence of visual impairment on quality of life [26, 27]. For the purposes of the current work, we focused on the following items: (1) overall health; (2) difficulty with peripheral vision; and (3) driving difficulties.

### Driving simulation

#### Driving simulator

SENSEI (Simulator for Ergonomics, Neuroscience, Safety Engineering and Innovation), a DriveSafety (Salt Lake City, UT) RS-600 Research Simulator, provided the driving environment [28]. SENSEI is constructed around a full-sized automotive cab (2004 Ford Focus). SENSEI’s cab is centered within 7 Ultra-HD (3840 × 2160) curved LED displays that provide a 290-degree panoramic field of view. Additional side and center rear-view LCD monitors provide a full 360-degree simulated environment. SENSEI is fully integrated with instrumentation for recording vehicle and driver performance measures.

#### Driving simulator task

Participants completed a concurrent driving simulator visual field (DSVF) task that we recently described [28]. Briefly, the DSVF maps out grid locations spanning 60° and 20° of total horizontal and vertical visual angle, respectively. Grid locations were tested in random patterns, and each grid location was tested 4 times. Patients were instructed to press a red button on the steering wheel after detecting a stimulus. Hits were counted if the button was pressed prior to a subsequent stimulus presentation. Each DSVF was approximately 4 min, and each DSVF procedure was repeated twice. Pass criterion, defined as greater than 50% detection rate, was chosen for its favorable sensitivity and specificity trade-off profile [29]. VFI calculations were performed using standard HVF construction methods [22]. Gray scales and DS-VFI estimates from the DSVF are comparable to those obtained in the clinic [28].

#### Driving simulation

Driving scenarios were completed on a straight two-lane rural road, with a 3.6-m lane width and 88.5 km/h speed limit. All drives were completed on a straight road without pedestrians, vehicles, or traffic lights. Vehicle performance data were continuously sampled at 60 Hz. Vehicle sensors continuously monitored vehicle speed, steering wheel position, and lateral and longitudinal acceleration. From these vehicle parameters we derived the following outcome measures: mean velocity, velocity variability, maximum velocity, percentage of drive that velocity exceeded the speed limit, steering wheel variability, lateral and longitudinal acceleration variability. These measures track vehicle control with respect to speed and lane position, which are predictors of driving safety [30–32].

### Statistical analysis

Statistical analyses were performed with SAS software version 9.4 (SAS Institute Inc., Cary NC). Categorical data were descriptively summarized using frequencies and percentages tables. Numeric data were descriptively summarized using means and standard deviations. Univariate graphs were created for continuous variables to investigate distributional properties. Independent-sample t-tests were performed to assess between-group differences in continuous variables (e.g. age, VFI, RNFL thickness); Welch-Satterthwaite t-tests were used when variance was unequal between groups. Wilcoxon rank sum test was used to test for differences in MoCA total score and subscales between groups. Chi-squared tests were performed to assess between-group differences in gender. Linear models and Spearman correlations were used to determine associations between neurocognitive performance and with clinical characteristics. Linear mixed models were constructed to determine whether driving performance was associated with clinical characteristics. Model assumptions were assessed using residual plots, and natural logarithmic transformations were applied to outcome variables as needed. For the multivariable models, a logit transformation was applied OU values to expand the upper range of data values, and better meet the model assumptions. Where appropriate, Kenward-Roger degrees of freedom corrections were used to account for missing data [33]. Statistical significance was set to the standard *p* < 0.05 level. We also estimated standardized effect sizes [34] (Cohen’s d) for main effects and reported those that exceeded a medium effect size of 0.5.

## Results

### Clinical characteristics

A total of 43 patients were recruited from Truhlsen Eye Institute at UNMC and enrolled into the pilot study (Table 1). Glaucoma patients (69.8 ± 11.3; *n* = 25) were significantly older than glaucoma suspects (60.3 ± 13.1; *n* = 18). We therefore included age as a separate factor in our statistical models.

**Table 1:** Clinical Characteristics

MEASURE	ALL (***n*** = 43)	SUSPECT (***n*** = 18)	GLAUCOMA (***n*** = 25)	Test statistic, df	***p***-value
Age, years	65.8 ± 12.8	60.3 ± 13.1	69.8 ± 11.3	2.54, 41	0.015
Gender					
Female	25 (58%)	14 (78%)	11 (44%)	4.91, 1	0.027
Male	18 (42%)	4 (22%)	14 (56%)		
VA OD	0.09 ± .11	0.05 ± .07	0.12 ± 0.13	2.53, 38.9	0.016
VA OS	0.11 ± .12	0.08 ± 0.12	0.13 ± 0.12	1.50, 41	0.14
VA Worst	0.10 ± 0.11	0.06 ± 0.09	0.13 ± 0.12	2.03, 41	0.049
MD OD	−4.97 ± 7.54	0.25 ± 0.94	−8.73 ± 8.00	4.72, 41	<.0001
MD OS	−5.84 ± 7.93	−0.23 ± 0.95	−9.87 ± 8.30	4.89, 41	<.0001
MD Worst	−7.02 ± 8.71	−0.01 ± 0.94	−12.08 ± 8.29	6.13, 41	<.0001
VFI OD	85.6 ± 22.2	99.7 ± 0.6	75.4 ± 24.7	4.91, 24.0	<0.0001
VFI OS	84.4 ± 23.8	99.6 ± 0.6	73.5 ± 26.4	4.95, 24.0	<0.0001
VFI Worst	80.3 ± 25.8	99.4 ± 0.7	66.6 ± 26.3	6.24, 24.0	<0.0001
VFI OU	85.6 ± 18.3	98.1 ± 1.2	77.1 ± 19.7	4.99, 21.2	<0.0001
gRNFLT Worst	73.2 ± 22.5	88.2 ± 19.2	59.8 ± 15.9	5.00, 36	<0.0001
gRNFLT Best	80.6 ± 19.5	93.6 ± 9.8	68.9 ± 18.7	5.18, 29.4	<0.0001
VFQ Total	89.3 ± 7.4	91.6 ± 7.6	87.8 ± 7.0	1.60, 35	0.12
VFQ Peripheral	87.8 ± 15.2	93.3 ± 11.4	84.1 ± 16.4	1.88, 35	0.068
VFQ Driving	83.7 ± 14.4	87.8 ± 15.1	80.9 ± 13.5	1.46, 35	0.15

(*VA* Visual Acuity, *OD* right eye, *OS* left eye, *OU* both eyes, *MD* mean deviation, *VFI* visual field index, *gRNFLT* global retinal nerve fiber layer thickness, *VFQ* visual function questionnaire)

Mean ± SD

We evaluated between-group differences across clinical characteristics. Results are summarized in Table 1. In the glaucoma group, OU VFI estimates varied from 24 to 98%, representing a broad range of binocular visual field defects. MD was lower for patients than suspects in OD (t _(41)_=4.72, *p* < .0001), OS (t _(41)_=4.89, *p* < .0001), and worst eye (t _(41)_=6.13, *p* < .0001). RNFL thickness was lower for patients than suspects in both best (t_(29.4)_ = 5.18, *p* < 0.0001; Cohen’s d = 1.65) and worst (t _(36)_=5.0, *p* < 0.0001; Cohen’s d = 1.61) eye. NEI-VFQ outcomes revealed a non-significant difference in visual quality of life in patients relative to suspects on peripheral (t _(35)_=1.88, *p* = 0.068; Cohen’s d = 0.65) scores, and no difference in global or driving scores (*p* > 0.10; Cohen’s d < 0.53). Worst eye VFI indices were associated with worst eye RNFL thickness (β = 0.64 ± 0.13; F _(1, 34)_=22.80, *p* < 0.0001; Cohen’s d = 1.61) and age (β = − 0.66 ± 0.26; F _(1, 34)_=6.39, *p* = 0.016; Cohen’s d = 0.85).

### Neurocognitive performance

There were no significant between-group differences in MoCA total (*p* = 0.58) or sub-scores (*p* > 0.16; Cohen’s d < 0.49; Table 2). Multivariate linear regression was used to determine whether any clinical characteristics were associated with MoCA performance. MoCA total scores were significantly associated with OU VFI estimates (β = 9.69 ± 3.28; F _(1, 26)_=8.72, *p* = 0.0066; Cohen’s d = 1.14). MoCA total scores were not significantly associated with RNFL thickness (*p* = 0.20), age (*p* = 0.86), gender (*p* = 0.87), or visual acuity (*p* = 0.83).

**Table 2:** Neurocognitive Performance

MEASURE	ALL (***n*** = 43)	SUSPECT (***n*** = 18)	GLAUCOMA (***n*** = 25)	***p***-value*
MoCA – Visuo-Spatial	3.8 ± 1.0	3.9 ± 0.8	3.6 ± 1.2	0.52
MoCA – Naming	3.0 ± 0.2	2.9 ± 0.3	3.0 ± 0	0.098
MoCA – Attention	5.3 ± 0.8	5.2 ± 0.9	5.4 ± 0.7	0.49
MoCA – Language	2.4 ± 0.8	2.5 ± 0.9	2.3 ± 0.8	0.36
MoCA – Abstraction	1.5 ± 0.6	1.7 ± 0.5	1.4 ± 0.7	0.27
MoCA – Verbal Recall	3.0 ± 1.7	3.2 ± 1.2	2.8 ± 2.0	0.86
MoCA – Total Score	25.0 ± 2.7	25.4 ± 2.4	24.7 ± 3.0	0.58

*Wilcoxon rank sum test

We examined which neurocognitive functions were specifically associated with OU VFI and RNFL thickness measurements. Visuo-spatial and executive function abilities were significantly associated with OU VFI (Spearman *ρ* = 0.41, *p* = 0.012). Clinical characteristics and MoCA naming, attention, language, abstraction, verbal recall, and orientation scores were not.

### Driving simulator performance

Driving simulator adaptation syndrome (or simulator sickness) equally affected glaucoma patients (12%) and suspects (17%) (χ^2^ = 1.01, *p* = .32). We observed significant between-group differences in steering wheel variability (*p* = 0.0001) and lateral acceleration variability (*p* < 0.0001), indicating glaucoma patients, relative to suspects, showed greater variability in lateral vehicle position and lane maintenance in the driving simulator (Table 3). No other driving simulator performance measures revealed significant between group differences.

**Table 3:** Driving Performance

MEASURE	SUSPECT (***n*** = 15)			GLAUCOMA (***n*** = 22)			
	Mean	Lower 95% CI	Upper 95% CI	Mean	Lower 95% CI	Upper 95% CI	*p*-value
Steering Wheel Variability*	0.219	0.140	0.342	0.754	0.521	1.090	0.0001
Lateral Acceleration Variability*	0.0014	0.0009	0.0023	0.0052	0.0036	0.0076	<0.0001
Mean Velocity	24.76	24.02	25.50	24.93	24.32	25.55	0.72
Velocity Variability*	1.549	1.100	2.182	1.622	1.218	2.161	0.84
Longitudinal Acceleration Variability*	0.0155	0.0104	0.0230	0.0250	0.0180	0.0347	0.067
Overlimit 55 mph (%)	0.549	0.411	0.687	0.600	0.490	0.711	0.56
Overlimit 60 mph* (%)	0.077	0.041	0.145	0.152	0.087	0.263	0.11
Maximum Velocity	27.71	26.74	28.69	27.86	27.05	28.66	0.82

*Lognormal distribution back transformed to original scale for reporting

We used modeling procedures to determine whether driving performance measures were associated with any clinical characteristics (Table 4). We found steering wheel variability (log scale) was associated with logit of OU-VFI (β = − 0.19 ± SE = 0.067; *p* = 0.0069), and VFQ Driving sub-score (β = − 0.031 ± 0.0098; *p* = 0.0037), but not age (*p* = 0.56), visual acuity (*p* = 0.46), or MoCA total score (*p* = 0.24). We found that lateral acceleration variability (log scale) was associated with logit of OU-VFI (β = − 0.20 ± 0.069, *p* = 0.0064) and VFQ Driving sub-score (β = − 0.029 ± 0.010; *p* = 0.0063), but not age (*p* = 0.49), visual acuity (*p* = 0.42), or MoCA total score (*p* = 0.17).

**Table 4:** Multivariable model of vehicle control measures (natural log scale) with OU variable

Vehicle Control Measure	Effect	Estimate	SE	DF	F Value	***p***-value
Steering Wheel Variability	Intercept	3.1881	1.5922			
	Age	0.0141	0.0105	30.76	1.34	0.1906
	VA_Worst	−1.9879	1.2156	31.32	− 1.64	0.112
	Logit (OU)	−0.1875	0.0666	52.25	−2.81	0.0069
	MoCA_Total Score	− 0.0632	0.0527	32.1	−1.2	0.2393
	VFQ_Driving	−0.0308	0.0098	30.56	−3.15	0.0037
Lateral Acceleration Variability	Intercept	−1.6206	1.6285			
	Age	0.0144	0.0108	30.8	1.33	0.1917
	VA_Worst	−1.9368	1.2439	31.33	−1.56	0.1295
	Logit (OU)	−0.1966	0.0691	50.23	−2.85	0.0064
	MoCA_Total Score	−0.0753	0.0540	32.06	−1.39	0.1727
	VFQ_Driving	−0.0293	0.0100	30.61	−2.93	0.0063

Next, we sought to determine if model outcomes would change if glaucoma group, rather than OU-VFI, were used as the measure of disease burden (Table 5). We found steering wheel variability (log scale) was associated with glaucoma group (*p* = 0.0003), MoCA total score (β = − 0.11 ± 0.05; *p* = .028), VFQ Driving sub-score (β = − 0.026 ± 0.009; *p* = 0.0087), but not age (*p* = 0.61) or visual acuity (*p* = .08). We found that lateral acceleration variability (log scale) was associated with glaucoma group (*p* = 0.0002), MoCA total score (β = − 0.12 ± 0.047; *p* = .014), VFQ Driving sub-score (β = − 0.024 ± 0.009; *p* = 0.014), but not age (*p* = 0.63) or visual acuity (*p* = .089).

**Table 5:** Multivariable model of vehicle control measures (natural log scale) with glaucoma group variable

Vehicle Control Measure	Effect		Estimate	SE	DF	F Value	***p***-value
Steering Wheel Variability	Intercept		3.3145	1.4752			
	Age		0.0052	0.0102	31	0.51	0.6105
	VA_Worst		−2.0259	1.1191	31	−1.81	0.0800
	MoCA_Total Score		−0.1078	0.0466	31	−2.31	0.0276
	VFQ_Driving		−0.0258	0.0092	31	−2.8	0.0087
	Group	Glaucoma vs. Suspect	1.0911	0.2667	31	4.09	0.0003
Lateral Acceleration Variability	Intercept		−1.4902	1.4858			
	Age		0.0050	0.0103	31	0.49	0.6287
	VA_Worst		−1.9820	1.1272	31	−1.76	0.0885
	MoCA_Total Score		−0.1220	0.0470	31	−2.6	0.0142
	VFQ_Driving		−0.0240	0.0093	31	−2.59	0.0144
	Group	Glaucoma vs. Suspect	1.1516	0.2686	31	4.29	0.0002

## Discussion

We studied the association between binocular visual field loss, global neurocognitive performance, and quantitative measures of simulated motor vehicle control in drivers diagnosed with glaucoma. Glaucoma drivers with a range of binocular visual field loss, relative to suspects without visual field loss, showed significantly worse lane position control as indexed by greater than a three-fold increase in lateral acceleration and steering wheel variability. Binocular visual field loss, neurocognitive performance, and self-reported driving performance were independently associated with measures of lane position control. Specifically, greater lateral acceleration variability was associated with: (1) greater binocular visual field loss; (2) worse neurocognitive performance; and (3) worse self-reported driving performance. Together, this work adds to our understanding of how driving behaviors, such as motor vehicle control, may contribute to motor vehicle crash risk in glaucoma by establishing a previously unexplored link between on-road driving results showing an association between binocular visual field loss and subjective qualitative ratings of lane maintenance errors [11], on the one hand, and driving simulator [13] and real-world driving [6] results showing an association between binocular visual field loss and simulated crash risk, on the other hand. Furthermore, this work demonstrates non-overlapping contributions of binocular visual field loss and global neurocognitive performance to driving performance in glaucoma.

Previous driving simulator studies have demonstrated increased simulator crash risk in glaucoma drivers [13, 14]. Szlyk and colleagues (2005) found increased simulated crash risk was associated with greater binocular visual field loss [13], where 57% of glaucoma drivers with less than 10 degrees of total peripheral visual field were involved in simulator accidents. Using glasses to artificially constrict concentric vision, Udagawa and colleagues showed that the number of driving simulator accidents was significantly higher in drivers with vision constricted to 10 and 15 degrees of visual angle, where greater visual constriction was associated with higher crash rates with vehicles approaching from the visual periphery [35]. Kwon and colleagues demonstrated increased crash risk in glaucoma drivers with binocular visual field loss in upper, lower, and left – but not right – visual fields [6], though how visual field loss contributed to crashes remains to be determined. Together, these results demonstrate that peripheral vision loss impedes detection of peripheral roadway objects, delaying object processing and resulting in increased risk in failing to avoid a motor vehicle crash. Interestingly, however, glaucomatous visual field loss does not delay detection of medium-to-high contrast peripheral visual stimuli during a driving simulator divided attention task [36]. By contrast, mechanisms explaining the relationship between peripheral visual field loss and predictors of increased crash risk, such as motor vehicle control, remain unknown. Drivers with severe peripheral visual field loss may be less able to track lane boundaries and maintain continuous control over lane position, which could explain increased simulator crash risk. In the current driving simulator study, we found greater binocular visual field loss was associated with greater lateral acceleration and steering wheel variability on a straight hazard-free highway. To further explore mechanisms explaining the putative relationship between poor motor vehicle control and increased crash risk in glaucoma, it will be necessary to demonstrate: (1) poor lane maintenance control mediates the relationship between binocular visual field loss and simulator crash risk; (2) greater binocular visual field loss is associated with perceptual deficits in lane boundary detection; and (3) improving lane boundary detection decreases simulator crash risk.

Driving simulators offer a safe environment for exploring mechanisms of driving safety risk. To evaluate their external validity, however, results from the current driving simulator study must be translated to on-road driving studies [6–8, 15]. Wood and colleagues (2016) reported poorer subjective ratings of lane positioning in glaucoma driver relative to controls [37], though the relationship between lane position and binocular visual fields was unexplored. Bowers and colleagues (2005), by contrast, found that more restricted binocular visual fields were associated with subjective ratings of on-road lane maintenance errors during curve taking [11], suggesting our results may translate to on-road driving performance. Translating simulated and on-road driving performance, however, demands similar comparisons. Quantitatively evaluating on-road lane maintenance control measures, such as those reported here, has the additional benefit of automation to facilitate the rapid analysis of large driving datasets collected from instrumented vehicles equipped with remote sensors. To further explore the external validity of current driving simulator results to on-road driving performance and increased crash risk in glaucoma, further work is need to demonstrate: (1) greater binocular visual field loss is associated with quantitative measures of poor lane maintenance during an on-road drive in a remotely instrumented vehicle; (2) quantitative measures of poor on-road lane maintenance mediate the relationship between binocular visual field loss and motor vehicle crash rates as indexed by state records or long-term remote monitoring via real-world driving recorders; and (3) an association between simulated and on-road quantitative measures of poor lane maintenance.

Information processing models of driving performance are essential to understanding mechanisms of driving safety risk in medical populations [38]. According to information processing models, neurocognitive functions are downstream of, and consequently impacted by, sensory function. Numerous studies have demonstrated how sensory dysfunction in glaucoma (i.e. peripheral visual field loss) contributes to driving performance and crash risk [6–8, 15]. In healthy drivers, contributions of neurocognitive functions to driving safety risk are well-documented [10, 39, 40]. To our knowledge, the current study is the first to explicitly evaluate the association between neurocognitive function and driving performance in glaucoma drivers. The handful of studies that have included neurocognitive measures in their study design relied on dementia screening tools that were utilized to statistically adjust for mental status without describing its contribution to driving performance [6, 8]. Tatham and colleagues evaluated performance during a novel divided attention task that required detecting a variable contrast peripheral visual stimulus during a concurrent curve negotiation or car following driving task load in a simulator [36]. Divided attention task performance was associated with concurrent car following or curve negotiation performance, visual field loss, and RNFL thickness, but not MoCA performance. Further, the authors found no difference in MoCA performance between glaucoma and control groups, which we replicated here, but associations between MoCA and driving performance were unexplored. In the current work, we found binocular visual field loss and global neurocognitive performance independently predicted simulated motor vehicle control performance, suggesting sensory and neurocognitive functions are both contributing factors to information processing models of driving safety risk in glaucoma. Indeed, emerging evidence shows that glaucoma patients are at risk for neurocognitive impairment [41–43] that may be explained by brain network dysfunction in non-visual brain regions [44, 45]. Accounting for neurocognitive performance in future studies will be important to further our understanding of increased crash risk in glaucoma.

Comparisons between simulated and on-road driving performance are limited by several factors, including the extent to which: (1) drivers suspend disbelief and immerse themselves in the simulated environment; and (2) the simulated environment replicates the complexity of on-road sensory experiences and roadway vehicle behaviors. Due to these factors, some drivers may be less likely to take driving simulations seriously, more likely to engage in more risky driving behavior, or not fully replicate their naturalistic driving behavior. Thus, driving simulator study results, such as those reported here, must be evaluated in the context of these limitations. Despite these limitations, driving simulators are a safe and externally valid alternative to on-road vehicles for studying driving behavior [12]. Future applications of this research will benefit from exploring the current pattern of results during on-road driving performance.

The current study must be considered in the context of its limitations. (1) Study results were obtained from a relatively small sample size. Due to the limited study power, other factors and co-morbidities associated with driving (e.g. manual dexterity, fatigue) could not be explored. A larger study that replicates current study findings and includes additional factors will be necessary. (2) Glaucoma suspects were recruited as the control group because they present with the same pathophysiologic risk factors of glaucoma without visual field loss. A proportion of glaucoma suspects, including those enrolled in the current study, will progress to a glaucoma diagnosis with visual field loss. In future studies, enrolling an additional healthy control group will provide further support that findings reported here are related to visual field loss rather than glaucoma-related risk factors. (3) Glaucoma patients were statistically older than glaucoma suspects, suggesting key study findings may be interpreted as an age-related, rather than glaucoma-related, difference in simulated driving performance. Ruling out this alternative explanation, key findings remained when age was included as a separate factor in regression analyses. In future studies, it will be important to match comparison groups on age, among other demographic factors (4) Study results contribute to our mechanistic understanding of simulated driving performance in glaucoma drivers. To determine the external validity of these findings, driving simulator results demand translation to on-road driving performance. (5) MoCA was included in this study as a global measure of neurocognitive function. To better understanding which specific neurocognitive functions predict driving performance in glaucoma, more comprehensive neurocognitive assessments will be necessary in future studies.

## Conclusions

In conclusion, poor driving simulator vehicle control was independently associated with greater visual field loss and worse neurocognitive performance, suggesting both factors play a major role in information processing models of driving performance in glaucoma. These results suggest that both visual field loss and neurocognitive function must be considered when developing models of poor vehicle control in glaucoma. It will be important to establish the external validity of current study results by demonstrating a relationship between simulated and on-road driving performance.

## Abbreviations

DSVF: Driving simulator visual field
HVF: Humphrey visual field
MOCA: Montreal Cognitive Assessment
NEI: National Eye Institute
OCT: Optical coherence tomography
OU-VFI: Binocular visual field index
RNFL: Retinal nerve fiber layer
SENSEI: Simulator for Ergonomics, Neuroscience, Safety Engineering and Innovation
TD: Total deviation
TV: Threshold value
UFOV: Useful field of view
UNMC: University of Nebraska Medical Center
VF: Visual field
VFI: Visual field index
VFQ: Visual function questionnaire
